# Rapid response teams: how are they best used?

**DOI:** 10.1186/s13054-016-1425-z

**Published:** 2016-08-19

**Authors:** Thomas Rozen, Warwick Butt

**Affiliations:** 1 Intensive Care Unit, Royal Childrens Hospital, Melbourne, Australia; 2 Murdoch Childrens Research Center, Melbourne, Australia; 3 Department of Paediatrics, University of Melbourne, Melbourne, Australia

*Never discourage anyone … who continually makes progress, no matter how slow.* Plato

Many cardiac arrests in hospitalized patients are preceded by warning signs such as derangements in vital signs [1–3]. Despite advancements in many aspects of health care, in-hospital cardiac arrests continue to have a mortality of approximately 80 % [4]. Efforts to reduce mortality in hospitalized patients therefore include a focus on the deteriorating patient in order to provide earlier treatment and prevent further deterioration and ultimately cardiac arrest. Serious adverse events (SAEs) unrelated to the admission diagnosis and due to incorrect medical management may occur in up to 17 % of hospital admissions, and may result in prolonged length of hospital stay, permanent disability, or even death [5]. The rapid response team (RRT) is tasked with preventing or responding to SAEs. Two-tiered systems involving RRTs as distinct from cardiac arrest or “code blue” teams have been implemented in many hospital settings in most countries. These systems aim to identify and manage patients at high risk of further deterioration, altering their trajectory and improving morbidity and mortality outcomes.

The rapid response system describes the hospital-wide approach to recognizing and treating deterioration, and includes an afferent limb (trigger), an efferent limb (RRT), administration, and governance [6]. Ideally, the afferent limb and triggering mechanism would identify only those patients likely to benefit from intervention; however, no such criteria currently exist. “Crisis detection” therefore may be from chart-based predefined observation cutoff points in single or multiple vital signs, early warning scoring systems, computer algorithm-based warnings, or clinical concern. The efferent or responder limb may have various compositions including a critical care trained nurse and/or doctor, although a recent meta-analysis found that the presence of a physician in the rapid response system was not significantly associated with mortality reduction [7].

While multiple prior single-center studies have shown a reduction in rates of cardiac arrest, to date there has been only one large multicenter randomized controlled trial, the Medical Early Response Intervention and Therapy (MERIT) study, which failed to demonstrate an improvement in the Australian setting in cardiac arrest, unplanned ICU admission, or unexpected death despite greatly increased emergency team calling [8]. As a consequence of the significant resource requirements to function effectively, these systems are costly; and despite more than 27 published studies to date of mostly uncontrolled trials of implementation, it remains controversial whether rapid response systems are effective at preventing unexpected deaths [9]. Nevertheless, many affluent nations have begun to introduce these systems.

The Cost and Outcomes of Medical Emergency Teams (COMET) study [10] evaluated the nationwide implementation of a rapid response system in the Netherlands in 2015. This was a pragmatic before-and-after implementation study involving 12 Dutch hospitals, more than 160,000 patients, and more than a million inpatient days, with a significant improvement in the primary composite endpoint of cardiopulmonary arrests, unplanned ICU admissions, and all-cause mortality in patients in general hospital wards.

In the June issue of *Critical Care*, Brunsveld-Reinders et al. [11] present the findings of their post-hoc analysis of the COMET study [10]. Here they have substituted death without limitation of medical treatment (LOMT) or “unexpected death” for all-cause mortality, and studied the proportion of patients dying with a LOMT order and the timing and prevalence of LOMT with the introduction of a RRT.

Brunsveld-Reinders et al. report that the unadjusted OR for death without LOMT (“unexpected death”) was 0.557 (95 % CI, 0.40–0.78) while the originally reported unadjusted OR for all-cause mortality was 0.865 (95 % CI, 0.77–0.97). Furthermore, in 13 % of patients who died and for whom a RRT was called, a LOMT was instituted or changed after consultation of the RRT. In this study, 65 % of patients who died had a LOMT placed at admission while 85 % of patients who died had some form of LOMT present at the time of death. Therefore only 15 % died without a LMOT order. There were no statistically significant differences in the overall rates of LOMT orders after introduction of the rapid response system, and both before and after RRT implementation the last change to LMOT was in the final few days of the hospital stay.

There are substantial differences internationally in withholding or withdrawing life support [12]. Cultural differences in practices relating to limiting medical treatment will affect interpretation of cardiac arrest data and overall death rates, because the actions of medical staff will differ. Hence, it is unclear whether these findings would be reproducible in other countries with different practices relating to LOMT. In an analysis of 14,488 patients from 282 ICUs in seven different geographical regions, deaths occurred after a decision to limit treatment at varying rates depending on the region [13]. These ranged from 26 % of ICU patients in Central and South America compared with 48 % in central and Western Europe, and there was an even wider variation for individual countries. Similarly, in the End-of-Life Practices in European Intensive Care Units (ETHICUS) study which assessed ICU end-of-life care in European countries, the northern European group (Denmark, Finland, Ireland, the Netherlands, Sweden, and the UK) had more limitations, less CPR use, and less time until a limitation of treatment was determined [14]. Withdrawal of life-sustaining treatments was also more common (47 % vs 18 %, *p* < 0.001) than in southern Europe (Greece, Israel, Italy, Portugal, Spain, and Turkey).

While initially intended to prevent cardiac arrests, unexpected deaths, and unplanned admissions to the ICU, emerging evidence suggests that RRTs are also used to review patients who do not have reversible deterioration and are at the end of life [15]. Brunsveld-Reinders et al. [11] highlight the impact of RRT in patients where LOMT has and has not been implemented. Clearly there is an ongoing need for the intensive care community to advocate for early discussion about appropriate limitations of therapies and compassionate end of life care prior to the point of deterioration, while simultaneously working to achieve better methods of identifying those patients most likely to benefit from ICU interventions.

## Abbreviations

COMET, Cost and Outcomes of Medical Emergency Teams; CPR, cardiopulmonary resuscitation; ETHICUS, End-of-Life Practices in European Intensive Care Units; LOMT, limitation of medical treatment; MERIT, Medical Early Response Intervention and Therapy; OR, odds ratio; RRT, rapid response team; SAE, serious adverse event
